# Canine Soft Tissue Sarcomas: Can Being a Dog’s Best Friend Help a Child?

**DOI:** 10.3389/fonc.2017.00285

**Published:** 2017-11-23

**Authors:** Bernard Séguin

**Affiliations:** ^1^Flint Animal Cancer Center, Department of Clinical Sciences, Colorado State University, Fort Collins, CO, United States

**Keywords:** soft tissue sarcoma, dog, translational model, pediatric, adolescent, young adults

## Abstract

Soft tissue sarcomas (STSs) remain a therapeutic challenge for pediatric and adolescent and young adult (AYA) patients. Still today, surgery, radiation therapy, and chemotherapy remain the mainstay of treatment. Obstacles in developing new treatment approaches to improve the outcome are: few patients to enroll in clinical trials, and the diversity of tumor biology between histologic subtypes. Pet dogs may offer an additional strategy to discover and test new therapeutic avenues. The number of dogs diagnosed with a STS each year in the United States is estimated to be around 27,000 to 95,000. In comparison, approximately 900 children less than 20 years old and 1,500 AYAs between 15 and 29 years old are diagnosed with a STS each year in the United States. The mainstay for treatment of STSs in dogs is also surgery, with radiation therapy and chemotherapy when necessary. Similar to what is seen in humans, grade and stage are prognostic in dogs. In one comparative study of the histology and immunohistochemistry of canine STSs, most tumors were diagnosed as the human equivalent of undifferentiated sarcoma, spindle cell sarcoma, or unclassified spindle cell sarcoma. But much work remains to be done to fully assess the validity of canine STSs as a model. Gene expression analysis has been done in a limited number of canine STSs. Tissue banking, development of cell lines, and the ability to mobilize large-scale clinical trials will become essential in veterinary medicine to benefit both dogs and humans.

Soft tissue sarcomas (STSs) are a large and complex family of tumors with unifying features. The first obvious one is their tissue of origin. They arise from the tissues that emerge from the embryonic mesoderm and therefore are all mesenchymal. The WHO defines STSs as soft tissue tumors with malignant potential, that is, a propensity for locally destructive growth, risk of recurrence, and risk of distant metastasis (1, 2).

Soft tissue sarcomas remain a therapeutic challenge for some pediatric and adolescent and young adult patients. Still today, surgery, radiation therapy, and chemotherapy remain the mainstay of treatment (2). For most STSs, complete histological resection with 1–2 cm margins is the primary treatment modality in humans (3). Complete resection in pediatric patients can lead to a cure rate of 85 versus 35% for those with incomplete margins (4). Radiation therapy can increase the cure rate close to 69% (4). First-line chemotherapy regimens for STSs usually consist of doxorubicin with or without ifosfamide. In patients with locally advanced, unresectable, or metastatic high-grade STS, median overall survival was 13 months with doxorubicin treatment and 14 months with doxorubicin and ifosfamide treatment in one study (5). It is clear that there is a subset of patients for whom current “traditional” therapies fail and therefore novel and improved therapies are required.

Obstacles in testing new treatment approaches to improve the outcome are few patients to enroll in clinical trials, and the diversity of tumor biology between histologic subtypes (2). Pet dogs may offer an additional strategy to discover and test new therapeutic avenues. STSs are relatively common in dogs and most frequently develop in the subcutaneous location (6). They represent between 9 and 15% of all subcutaneous and cutaneous tumors in dogs (6, 7). With the annual incidence of STSs being approximately 35–122 per 100,000 dogs (8–10) and with an estimated 78 million dogs owned in the United States (11), the number of dogs diagnosed with a STS each year in the United States is inferred to be 27,000 to over 95,000. In comparison, approximately 900 children less than 20 years old and 1,500 adolescents and young adults (AYAs) between 15 and 29 years old are diagnosed with a STS each year in the United States (2).

With that many more dogs diagnosed with a STS than children, pet dogs might represent a unique opportunity to advance our knowledge of STSs and discover new therapies. With the dog’s life span being substantially shorter, the ability to gather data and reach a conclusion regarding the efficacy of a treatment can be achieved in a significantly shorter time period.

Currently, dogs too are treated with surgery, radiation therapy, and sometimes chemotherapy. The primary treatment modality in dogs is also surgery when feasible. Because of the invasive nature of many STSs, curative-intent surgeries are aggressive. Typically, a wide or radical excision is necessary for a complete excision (6). Wide excisions include a 2–3 cm margin around the edges of the tumor and one fascial plane deep (6). STSs can require radical surgeries such as maxillectomy/mandibulectomy, orbitectomy, body wall resection, or hemipelvectomy. With complete excision, local recurrence in dogs is <5% (12, 13). Incomplete margins increase the risk of local recurrence by 10.5 times (12). The role of radiation therapy is arguably not as well established for STSs in dogs. When used in the adjuvant setting following incomplete excision, local recurrence rates vary between 17 and 43% (14, 15). Maximally tolerated dose chemotherapy using doxorubicin is typically reserved for dogs with high-grade STS but has not been found to improve survival (16). Metronomic chemotherapy has been evaluated to help prevent local recurrence (17).

But is the dog a good translational model? While some canine tumors have been described as a good translational model, with osteosarcoma being the classic example (18), substantially more work needs to be done to have a definitive answer for STSs. The effort however appears worthwhile. The strengths of the dog as a translational model are: (1) first, as previously mentioned, the ability to collect data at a faster pace; (2) the tumors in pet dogs arise spontaneously, which is unlike murine models where the tumors are induced, more often by injecting tumor cells. The cells previously grown *in vitro* may not fully represent the tumor they originated from. The conditions *in vitro* are significantly different than the original conditions *in vivo* and undoubtedly a selection of cells occurred *in vitro*. In the dogs, the tumors being spontaneous, they possess the full heterogeneity of a natural tumor; (3) the dogs have an unmodified immune system. The state of the immune system is considered natural. The immune system may be able to interact with some of the treatments and affect response, for better or worse; (4) pet dogs share our environment and to some extent our lifestyles. The conditions in which pet dogs live in are not tightly regulated as they are for rodents and therefore not as artificial. This might have an impact on the response to a treatment; and (5) the size of a dog makes it such that the instruments and supplies used to diagnose and treat them are the same as they are in children and young adults. The size of a Chihuahua dog is similar to a baby (as small as 3 pounds) and the size of a Great Dane or Mastiff is the same as an adult (150–230 pounds). When performing imaging, surgery, radiation therapy, or chemotherapy, for example, in dogs, the same instruments and supplies are used as for humans. Therefore, the same limitations exist for dogs as for humans that are related to these instruments and supplies.

For dogs, STSs have conventionally been grouped together because of their similar biologic behavior and shared prognostic factors (6, 8, 19). While individual STSs may be histologically distinct, it is also practical to classify them together because some can be difficult to tell apart using light microscopy alone (6, 8, 20). The shared biologic behavior is that they are locally expansile or infiltrative, but have low metastatic potential (8, 19). When they do metastasize, it is most commonly to the lungs and rarely to the regional lymph nodes (6, 12). A higher histologic grade and mitotic count have been associated with an increased metastatic risk in dogs (20). Histological grade is the most important prognostic factor in human STS and is likely one of the most reliable criteria to anticipate outcome postoperatively in dogs (6, 12, 13). Higher tumor grades are associated with more aggressive biologic behavior, which translates to higher rates of local recurrence, distant metastasis and shorter disease-free intervals (6, 12, 13, 20). Similarly and not surprisingly, given mitotic index is one of the criteria used to determine tumor grade, mitotic index also, but independently, provides prognostic information about an individual tumor. A high mitotic index has been associated with increased rates of tumor recurrence, higher rates of metastasis, and reduced overall survival (6, 12, 13, 20–23).

While the convention has been to group all STSs together in dogs because of a shared biologic behavior and prognosis, there is evidence that the histologic type can have a prognostic significance. In dogs, neurofibrosarcomas, fibrosarcomas, and myxomas appear to have higher rates of local recurrence, whereas perivascular wall tumors have a lower rate of local recurrence (20, 23–25). This is likely due to perivascular wall tumors generally being of lower grade and neurofibrosarcoma, fibrosarcoma, and myxoma generally being of higher grade (23, 25). In humans, there is evidence that individual histologic STS will exhibit differences in local invasiveness, metastatic potential, and recurrence (26, 27). It is also important to note that most studies on canine soft issue sarcomas typically exclude histiocytic sarcoma, lymphangiosarcoma, hemangiosarcoma, synovial cell sarcoma, leiomyosarcoma, and rhabdomyosarcoma because they arise from other anatomic locations or they exhibit more malignant biologic behavior (6, 8, 20). This obviously creates a bias in the results of the studies which is in contrast to the classification scheme for human STSs, which is comparatively more inclusive (28).

Another unifying feature is that most STSs have peripheral compression of their cells, creating a pseudocapsule. The histologic evaluation of the pseudocapsule in human STSs has demonstrated varying integrity of this pseudocapsule with penetration by the tumor through the pseudocapsule increasing with tumor grade. The peripheral growth pattern of the tumor has been described as either pushing or infiltrative (29). The pushing pattern shows no evidence of infiltration of the tumor through the pseudocapsule, whereas the infiltrative pattern shows a tumor contour that is poorly defined or the presence of satellite nodules. The pushing pattern is seen more commonly with low-grade tumors and can be seen in 18% of high-grade tumors (29). The pushing pattern is prognostic for lower likelihood of local recurrence (29, 30). This has not been evaluated in canine STS.

But in some respects, our knowledge of STSs in dogs is limited compared to that in humans. Arguably, the most flagrant limitation and difference is the classification of STSs. In humans, STSs are separated into over 50 different subtypes by histological and molecular classifications (1, 31–33). In 2013, an update of the World Health Organization classification of tumors of soft tissue and bone was published (1). In this latest classification, diagnostic designations such as hemangiopericytoma and so-called malignant fibrous histiocytoma have been eliminated and several new entities have been introduced for the first time in this volume (33). A World Health Organization classification of the mesenchymal tumors of the skin and soft tissues of domestic animals was published in 1998 (34). No revision of this document has been published since then. One of the most comprehensive reviews of the histological diagnoses of mesenchymal tumors of skin and soft tissues has been published in 2017 by one of the original authors of the 1998 WHO classification (19). One obvious contrast between human and canine histologic classification is the diagnosis of hemangiopericytoma. Whereas this diagnosis has been eliminated from the human classification (1, 33), this designation persists on the canine side (19). Ironically, this relatively common diagnosis in dogs was given the name hemangiopericytoma because of some minor histological similarities to the tumor in humans (for which the designation hemangiopericytoma no longer exists) (19). However, the actual gross and histological features of the human tumor do not resemble those of the canine tumor (19). It is now recognized that the diagnosis of hemangiopericytoma in dogs is not a single tumor but represents a spectrum of tumors arising from the various cells of the perivascular wall and adventia (19). Because immunohistochemistry is not routinely performed in the clinical setting, some authors prefer the term perivascular wall tumors to encompass all these tumors (19, 24). Another example of deficiency in the classification of canine tumors in the clinical setting is the difficulty to distinguish perivascular wall tumors and peripheral nerve sheath tumors. The tumors can be differentiated by electron microscopy or immunohistochemistry but these tests are not routinely done for clinical cases. The reason for not performing the additional diagnostic tests is that they are believed to have similar biologic behaviors and prognoses and therefore the distinction becomes clinically irrelevant (8, 19). The term chosen by the pathologist to designate such tumor then becomes more a matter of their training bias.

Another difference between the classification of canine and human STSs is the diagnosis of undifferentiated sarcoma. Whereas the diagnosis of undifferentiated sarcoma has been in use in veterinary medicine for a while (6, 35), this is a new category on the human side (32). But the term undifferentiated sarcoma is not included in the list of possible canine STSs by every author, creating inconsistencies even just within the veterinary classification (8, 19, 36). It remains unknown if this same diagnosis in a human specimen and canine specimen would actually represent a similar tumor in both species. To illustrate this point, one study evaluated 32 canine STSs to determine the diagnosis assigned in the human classification (7). All myxosarcomas diagnosed by the veterinary pathologist were classified as spindle cell sarcoma with myxoid features or myxofibrosarcoma by the human pathologist. One myxofibrosarcoma diagnosed by the human pathologist was classified as a fibrosarcoma by the veterinary pathologist. All liposarcomas diagnosed by the veterinary pathologist were also diagnosed as liposarcoma by the human pathologist but one liposarcoma diagnosed by the human pathologist was classified as a neurofibrosarcoma by the veterinary pathologist. Hemangiopericytomas diagnosed by the veterinary pathologist were classified as undifferentiated sarcoma, unclassified spindle cell sarcoma, spindle cell sarcoma of the fibroblastic/myofibroblastic type, or low-grade tumor difficult to classify by the human pathologist. Other undifferentiated sarcomas diagnosed by the human pathologist were classified as neurofibrosarcoma, fibrosarcoma, or malignant fibrous histiocytoma by the veterinary pathologist (7). All in all, the majority (69%) of canine STSs were given the corresponding human pathological diagnosis of undifferentiated sarcoma, spindle cell sarcoma, or unclassified spindle cell sarcoma (7). Although these different diagnoses were utilized in the study (7), many pathologists find these terms synonymous. This study highlights that while some canine STSs are likely to be diagnosed with the same name with the human classification, others do not and histologic diagnoses are inconsistent between the veterinary and human classifications. These disparities can be explained by differences in terminology and diagnostic schema between human and veterinary pathologists (7). Clearly, other features will be required to classify and correlate canine and human tumors and determine their likelihood to be relevant as a translational model.

The features that will likely be very helpful to correlate canine and human tumors are genetic aberrations. Included in the latest publication on the WHO classification of human tumors of soft tissues are molecular genetic and cytogenetic characterizations. The inclusion of these features has been credited for allowing “more reproducible diagnosis, a more meaningful classification scheme, and provides new insights regarding pathogenesis” (32). The latest WHO classification of soft tissue tumors on the human side demonstrates the growing importance of cytogenetic and molecular genetic characterization of these lesions in becoming a keystone for a meaningful classification system. Over the past 20 years, diagnosis of soft tissue tumors has become more reproducible and, consequently, will likely help to identify more precise treatment strategies to improve the outcome (32).

Thorough and widespread molecular genetic and cytogenetic characterization is missing for canine STSs. The equivalent pathognomonic *COL1A1-PDGFB* gene fusion found in human dermatofibrosarcoma protuberans was found in a dermatofibrosarcoma protuberans-like canine tumor, with the equivalent fusion found being *COL3A1-PDGFB* in the dog (37). Gene expression analysis has also been done on a limited number of canine STSs. In one study using quantitative nuclease protection assay to measure RNA expression of multiple genes in archived formalin-fixed paraffin-embedded canine STSs, E2F1, MDM2, MYC, and EGFR varied in expression levels, with E2F1 significantly upregulated in grade 3 compared to grade 2 and grade 1 tumors (38). In another study, the gene Sprouty2 demonstrated the greatest relative expression, with a 96-fold higher expression in metastatic compared to non-metastatic STSs (39). Gene expression profiles of 22 canine STSs were performed before and after the first hyperthermia treatment administered as an adjuvant to radiotherapy and results were deposited into GEO (accession number: GSE23380) (40).

In an elegant example on how the dog can serve as a translational model, the bispecific, antiangiogenic targeted toxin eBAT was evaluated (41). Studies have shown that eBAT simultaneously targets urokinase plasminogen activator receptor (PLAUR gene) and EGFR. In *in vitro* experiments, eBAT was shown to kill canine and human sarcoma cells. Using the most current bioinformatics TCGA database, the expression on of *EGFR* and *PLAUR* on 212 human sarcomas was explored. *EGFR* and *PLAUR* gene expression were detectable in 100% of samples regardless of sarcoma type with a variation in intensity. *EGFR* and *PLAUR* expression was also evaluated in 51 canine hemangiosarcomas and 31 canine osteosarcomas. Expression of both *EGFR* and *PLAUR* genes was detectable in all canine sarcomas, with hemangiosarcoma having higher levels of *PLAUR* mRNA, and hemangiosarcoma and osteosarcomas having approximately equivalent levels of *EGFR* mRNA, which paralleled results in human sarcomas. In a clinical trial, eBAT was used in 23 dogs with hemangiosarcoma and overall survival was improved. Those results support further translation of eBAT for human patients with sarcomas (41). Genome-wide microarray-based somatic DNA copy number profiling of 75 primary canine hemangiosarcomas has been performed demonstrating extensive heterogeneity in somatic DNA copy number aberrations (42).

To find out if the dog is a relevant translational model for STSs, important steps are required to achieve this goal:

(A) Develop large-scale tissue banking and establishment of canine tumor cell lines: at what is arguably the largest tumor tissue bank from a single site, the Flint Animal Cancer Center at Colorado State University had STS samples from 219 dogs as of September 2017. Of these, 157 samples were available in RNA snap, 139 were preserved in RNA later, and 139 had at least 1 fresh frozen tumor sample available. The Canine Comparative Oncology and Genomics Consortium (CCOGC) is another resource for collecting and preserving canine biospecimens. The CCOGC has been able to provide canine STS samples for studies and will undoubtedly continue to play a key role in collecting and preserving tumor samples.
(B) Perform large scale molecular genetic and cytogenetic analyses of the different canine histologic types and of different grades for each.
(C) Improve the classification of STSs in dogs, which will have to include genetic and cytogenetic data to better parallel the human classification and allow comparisons and pairing with human tumors.
(D) Determine the prognostic significance of this improved classification in dogs.

Arguably, the single most important bottleneck in achieving these goals thus far has been the limited funding. Financing these studies may turn out to be an investment in the longer run. If and when the dog has been deemed a relevant model, the pediatric and veterinary medical communities will collaborate closely to enable designing and conducting large scale clinical trials in dogs. The Comparative Oncology Trials Consortium (COTC) would likely play a major role. The COTC is an active network of 22 academic comparative oncology centers, which are managed by the NIH-NCI-Center for Cancer Research’s Comparative Oncology Program. Its mission is to design and execute clinical trials in dogs with cancer to assess novel therapies, with the goal to generate data to help development of their future use in human cancer patients.

Investigating STSs in dogs to a greater and deeper level than has currently been done to ascertain if the dog is a relevant translational model will undoubtedly benefit the dog as we will gain a better understanding of these tumors. Should the dog turn out to be a good to excellent translational model, then testing new therapies in dogs will become justified and children and AYAs are likely to benefit from this approach. And in testing new therapies, our pet dogs will also benefit from these clinical trials, not to mention their human owners. With approximately 44% of all households in the United States having a dog (11), this will be a substantial number of humans who will enjoy a healthier pet.

## Author Contributions

The author confirms being the sole contributor of this work and approved it for publication.

## Conflict of Interest Statement

The research was conducted in the absence of any commercial or financial relationships that could be construed as a potential conflict of interest.
